# Association of serum fibroblast growth factor 21 with kidney function in a population-based Chinese cohort

**DOI:** 10.1097/MD.0000000000028238

**Published:** 2021-12-17

**Authors:** Rui Zhang, Yufeng Li, Xianghai Zhou, Fang Zhang, Meng Li, Simin Zhang, Xiuying Zhang, Xin Wen, Linong Ji

**Affiliations:** a Department of Endocrinology and Metabolism, Peking University People's Hospital, Xicheng District, Beijing, China; b Department of Endocrinology and Metabolism, Beijing Pinggu Hospital, Pinggu District, Beijing, China.

**Keywords:** eGFR decline, fibroblast growth factor 21, kidney function, metabolic disorder, progression

## Abstract

Fibroblast growth factor 21 (FGF21) plays a role in kidney disease. Circulating FGF21 levels are associated with kidney function and progression in patients with type 2 diabetes (T2D). However, the association between FGF21 and kidney function in the general population is still lacking. The aim of this study was to determine the association between FGF21 and kidney function and its progression in a Chinese cohort.

A total of 2425 participants from a population-based survey of diabetes and metabolic syndrome in Pinggu, Beijing, were included in the baseline analysis. After a median follow-up of 12 months, 2402 participants with baseline estimated glomerular filtration rate (eGFR) ≥60 mL/min/1.73 m^2^ were analyzed in the longitudinal study. The progression of kidney function was defined as an eGFR decline exceeding 3.3% per year. Serum FGF21 levels were measured using an enzyme-linked immunosorbent assay at baseline.

Male sex, body mass index (BMI), homeostasis model assessment of insulin resistance, higher levels of low-density lipoprotein cholesterol (LDL-c), uric acid, and FGF21 were associated with increased odds of a lower eGFR at baseline. The association of FGF21 with lower eGFR was independent of all the potential confounders in multivariable logistic regression (odds ratio, 1.005; 95% confidence interval 1.002–1.008). However, FGF21 was not associated with eGFR decline in the longitudinal analysis (odds ratio, 1.000; 95% confidence interval 0.998–1.001).

Increased serum FGF21 levels were independently associated with lower eGFR in this nonmedicated general population. FGF21 could be a biomarker of kidney function in the general population.

## Abstract 2

Supplemental Digital Content is available in the text

## 1 Introduction

Fibroblast growth factor 21 (FGF21) is a member of the endocrine subfamily of fibroblast growth factors that regulates the metabolism of glucose and lipids.^[1]^ The administration of FGF21 has demonstrated beneficial effects in dyslipidemia, obesity, and nonalcoholic fatty liver disease in both animal studies and human clinical trials.^[1–3]^ However, circulating levels of FGF21 were found to be paradoxically increased in individuals with multiple metabolic disorders, including obesity and type 2 diabetes (T2D).^[1,4]^ This paradox has usually been assumed to be a state of “FGF21 resistance” or a compensatory protective response to metabolic stress.

The association between FGF21 and kidney disease has also been a concern in recent years. There are inconsistent results in studies with different populations and study designs. A compensatory elevation of circulating FGF21 was observed in patients with chronic kidney disease (CKD) and diabetic nephropathy in cross-sectional studies.^[5]^ Longitudinal studies have suggested a predictive effect of higher levels of FGF21 on the decline of renal function or an increase in albuminuria in patients with T2D.^[6–8]^ Conversely, in a longitudinal study of community-dwelling individuals without apparent cardiovascular disease, the predictive effect was not confirmed.^[9]^

However, the mechanism involved in the association between FGF21 and kidney function remains unclear. Some studies^[10,11]^ suggested that FGF21 could protect against renal fibrosis, which is often found in patients with CKD.

If circulating FGF21 is an appropriate biomarker for kidney function or its progression in the general population, it is still uncertain. Therefore, we conducted the current observational study in a Chinese cohort derived from a general population unselected for existing diseases to evaluate the correlation between circulating FGF21 levels and kidney function, and to investigate the association of FGF21 with the progression of kidney function decline.

## 2 Materials and methods

### 2.1 Study population

**Figure 1:**
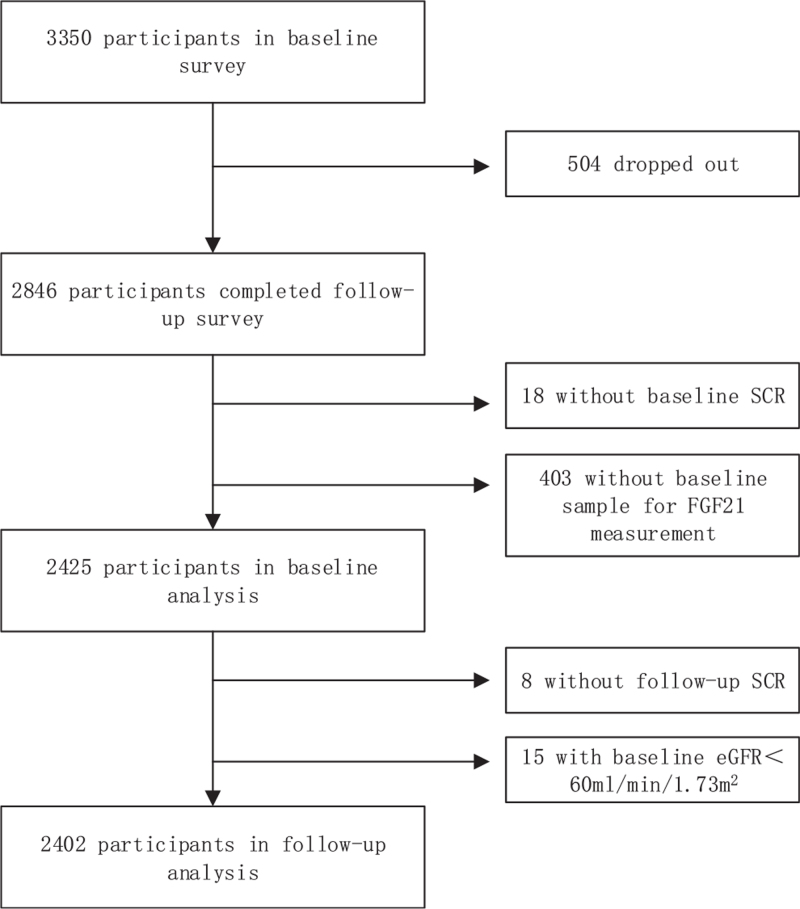
A flow diagram of the participants in baseline and follow-up analysis. eGFR = estimated glomerular filtration rate, SCr = serum creatinine.

This study contained both cross-sectional and longitudinal parts (Fig. 1). The participants in this study were enrolled from a population-based survey of diabetes and metabolic syndrome from March 2012 to May 2013 in the Pinggu District, which is located northeast of Beijing. Residents aged 25 to 74 years’ old and living in Pinggu for at least 5 years were included. A stratified random 2-stage cluster sampling process was used to recruit participants. The details of the sampling method and study population have been described previously.^[12]^ The baseline survey was approved by the Ethics and Human Subject Committee of the Peking University People's Hospital, and the follow-up survey (Pinggu Metabolic Disease Study) was approved by the ethics committee of the Peking University Medical Center. All participants provided written informed consent, and all methods were performed in accordance with the relevant guidelines and regulations.

A total of 3350 participants were included in the baseline survey. After a median follow-up of 1 year, 2846 participants (85.0%) completed the follow-up survey. Among them, 18 individuals without baseline serum creatinine (SCr) levels were excluded. A total of 403 participants without valid serum samples for FGF21 measurements were also excluded. Thus, 2425 participants were assessed in the baseline study. At the follow-up analysis, 8 individuals without follow-up SCr values and 15 individuals with baseline estimated glomerular filtration rate (eGFR) <60 mL/min/1.73 m^2^ were excluded. Finally, 2402 participants were included in the longitudinal analysis.

### 2.2 Data collection and biochemical measurements

Both baseline and follow-up data were collected by field researchers using a standard questionnaire. Anthropometric data, including height and weight, were measured. Body mass index (BMI) was calculated as weight (kg)/height^2^ (m^2^). Systolic blood pressure (SBP) and diastolic blood pressure were measured using an electronic sphygmomanometer (Omron, China). The 75-gram oral glucose tolerance test (OGTT) was performed for all participants without known diabetes in both the baseline and follow-up surveys. Patients with self-reported diabetes were tested only for fasting plasma glucose (FPG). According to OGTT, diabetes was diagnosed as FPG ≥7.0 mmol/L or 2 hours post-load plasm glucose ≥11.1 mmol/L.^[13]^

Plasma glucose was measured using the hexokinase method (COBAS C702, Roche Diagnostics, Tokyo, Japan). Hemoglobin A1c (HbA1c) was quantified using high-performance liquid chromatography (model ADAMS A1c HA-8160 chromatograph; Arkray, Inc., Kyoto, Japan). Alanine transaminase (ALT), aspartate aminotransferase (AST), SCr, uric acid (UA), total cholesterol, triglycerides, high-density lipoprotein cholesterol, and low-density lipoprotein cholesterol (LDL-c) were measured using an automated biochemical instrument (model Coulter UniCel DxC 800, Beckman, Miami, FL). Serum insulin levels were measured using the electrochemical luminescence method (COBAS E411, Roche Diagnostics, Tokyo, Japan). Urine albumin and creatinine levels were measured using a spot urine sample by the immunoturbidimetric assay and Jaffe's assay (COBAS C311, Roche Diagnostics, Tokyo, Japan), respectively. The urine albumin-to-creatinine ratio (UACR) was calculated.

Serum FGF21 levels were measured using a commercially available enzyme-linked immunosorbent assay kit (ELISA, Cloud Clone Corp., Houston, TX) according to the manufacturer's instructions. Serum samples were stored at −80°C until used for the measurement of FGF21. The homeostasis model assessment (HOMA-IR) was used to measure insulin resistance, which was calculated as fasting insulin (mU/L) × fasting plasma glucose (mmol/L)/22.5. eGFR was calculated from SCr using the Chronic Kidney Disease Epidemiology Collaboration equation.^[14]^ In the longitudinal analysis, eGFR decline was defined as a decline in eGFR that exceeded 3.3% per year.^[15]^

### 2.3 Statistical analysis

Statistical analysis was performed using Statistical Product and Service Solutions (SPSS) for Windows (version 16.0; SPSS Inc., Chicago, IL). Continuous variables with a normal distribution are presented as mean ±standard deviation. Medians (interquartile range) were used for values without a normal distribution including FGF21; medians (interquartile range) were used. Categorical data are presented as a number (percentage) and compared using the *χ*^2^ test. Student *t* test was used to compare variables with a normal distribution. The nonparametric Mann–Whitney test was used to compare those without a normal distribution. Linear regression was performed to determine the correlation between FGF21 levels and baseline characteristics. A binary logistic regression analysis was performed with lower eGFR as the dependent variable and sex, age, FGF21, and other potential confounders as independent variables in the baseline analysis. Logistic regression was also used in the longitudinal analysis, with eGFR decline as the dependent variable to assess the possible risk factors. Odds ratios (ORs) and 95% confidence intervals (CIs) were reported. Quartiles of FGF21 levels were also used as a categorical variable to assess the association of FGF21 with eGFR and eGFR decline.

## 3 Results

### 3.1 Baseline study

Of the 2425 participants, 15 (0.62%) individuals had an eGFR <60 mL/min/1.73 m^2^. The participants were classified into 2 groups according to the median eGFR (108.8 mL/min/1.73 m^2^) at baseline. Anthropometric and biochemical characteristics were compared between the 2 groups (Table 1). The participants with lower eGFR were significantly older in age, with more males, higher BMI, higher blood pressure, higher HbA1c, FPG, triglyceride, LDL-c, UA, UACR, and higher prevalence of diabetes diagnosed by OGTT than those with higher eGFR. The index of HOMA-IR was also higher in the group with a lower eGFR than in the higher eGFR group. Fibroblast growth factor 21 levels were significantly higher in individuals with a lower eGFR than those with a higher eGFR, (97.8 [67.9–134.7] vs 73.4[53.5–99.2], *P* < .001).

**Table 1:** Baseline characteristics of a total of 2425 participants classified by the median of eGFR.

	Lower eGFR	Higher eGFR	*P*
N	1212	1213	
Age, y^∗^	56.6 ± 8.7	41.0 ± 8.6	<.001
Male, n (%)^†^	593 (48.9)	492 (40.6)	<.001
BMI, kg/m^2^^∗^	26.4 ± 3.7	25.8 ± 3.8	<.001
SBP, mmHg^∗^	134.1 ± 16.6	124.5 ± 15.9	<.001
DBP, mmHg^‡^	87.2 ± 11.2	82.6 ± 11.1	<.001
HbA1c (%)^∗^	5.8 ± 1.0	5.6 ± 0.9	<.001
FPG, mmol/L^∗^	6.1 ± 1.8	5.7 ± 1.5	<.001
Fasting insulin, mU/L^‡^	7.2 (4.5–11.2)	7.0 (4.7–10.8)	.854
HOMA-IR^‡^	1.9 (1.1–3.0)	1.8 (1.1–2.7)	.039
Diabetes, n (%)^†^	235 (19.4)	125 (10.3)	<.001
ALT, U/L^‡^	19 (16–26)	19 (14–27)	.217
AST, U/L^‡^	21 (19–25)	20 (17–24)	<.001
TC, mmol/L^‡^	5.0 (4.4–5.7)	4.7 (4.2–5.3)	<.001
TG, mmol/L^‡^	1.3 (0.9–1.9)	1.1 (0.7–1.7)	<.001
LDL-c, mmol/L^∗^	2.9 ± 0.8	2.7 ± 0.7	<.001
HDL-c, mmol/L^∗^	1.2 ± 0.3	1.2 ± 0.3	.625
UA, μmol/L^‡^	282 (240–337)	254 (209–315)	<.001
UACR, mg/g^‡^	7.3 (2.8–18.5)	5.0 (1.8–12.5)	<.001
SCr, μmol/L^‡^	63.0 (54.5–73.7)	51.4 (44.0–61.0)	<.001
FGF21, pg/mL^‡^	97.8 (67.9–134.7)	73.4 (53.5–99.2)	<.001

The participants were divided into lower and higher eGFR groups with the median value of eGFR in baseline (108.8 mL/min/1.73m^2^).

∗ Variables were presented as the mean ± standard deviation.

† Variables were presented as frequency (%).

‡ Variables were presented as the median (interquartile range). ALT = alanine aminotransferase, AST = aspartate aminotransferase, BMI = body mass index, DBP = diastolic blood pressure, eGFR = estimated glomerular filtration rate, FGF21 = fibroblast growth factor 21, FPG = fasting plasma glucose, HbA1c = glycated hemoglobin A1c, HDL-c = high-density lipoprotein cholesterol, HOMA-IR = homeostatic model assessment of insulin resistance, LDL-c = low-density lipoprotein cholesterol, SBP = systolic blood pressure, SCr = serum creatinine, TC = total cholesterol, TG = triglycerides, UA = uric acid, UACR = urinary albumin/creatinine ratio.

The correlation between FGF21 and the other variables was analyzed using linear regression, which is expressed in the supplementary table, http://links.lww.com/MD2/A753. Age, SBP, AST, UA, and LCL-c were positively correlated with FGF21 levels in the whole population (n = 2425) and nondiabetes group (n = 2065). SCr level was positively correlated with FGF21 levels in all 3 groups. Two hours post-load plasma glucose and UACR were associated with FGF21 only in subjects without diabetes (n = 2065). Interestingly, BMI, HbA1c, fasting insulin, ALT, and HOMA-IR were negatively correlated with FGF21 in 2425 participants at baseline (Supplementary file, http://links.lww.com/MD2/A753).

In the multivariable logistic regression analysis (Table 2), male sex, BMI, HOMA-IR, higher levels of LDL-c, UA, FGF21, and the largest quartile of FGF21 were all associated with the increased odds of a lower eGFR after adjusting for age and sex (model 1 in Table 2). The association of FGF21 with lower eGFR was still significant after adjusting for all potential confounders in model 2 (OR 1.005; 95% CI 1.002–1.008). The largest quartile of FGF21 was independently associated with a lower eGFR. The OR (95% CI) was 1.818 (1.290–2.562; model 2 in Table 2).

**Table 2:** Associating factors for lower eGFR at baseline (n = 2425).

	Model 1-OR (95% CI)	Model 2-OR (95% CI)
Age	1.225 (1.206–1.245)^∗^	1.240 (1.218–1.262)^∗^
Female vs male	0.547 (0.437–0.686)^∗^	1.040 (0.794–1.362)
BMI	1.075 (1.041–1.110)^∗^	1.018 (0.980–1.057)
SBP	0.999 (0.992–1.006)	
HbA1c	0.944 (0.844–1.055)	
HOMA-IR	1.146 (1.071–1.226)^∗^	1.087 (1.010–1.170)^∗^
TG	1.021 (0.939–1.111)	
LDL-C	1.185 (1.026–1.369)^∗^	1.065 (0.915–1.239)
UA	1.009 (1.008–1.011)^∗^	1.008 (1.007–1.010)^∗^
UACR	1.000 (0.999–1.001)	
FGF21	1.005 (1.003–1.008)^∗^	1.005 (1.002–1.008)^∗^
Quartiles of FGF21		
Q1 (FGF21 < 58.50)	1.000	1.000
Q2 (82.67 > FGF21 ≥ 58.50)	0.988 (0.725–1.345)	0.980 (0.710–1.351)
Q3 (117.28 > FGF21 ≥ 82.67)	1.265 (0.924–1.733)	1.258 (0.907–1.746)
Q4 (FGF21 ≥117.28)	1.948 (1.404–2.702)^∗^	1.818 (1.290–2.562)^∗^

Model 1: adjusted for age and sex. Model 2: adjusted for all the statistically significant variables in Model 1. Quartiles of FGF21 and FGF21 as a continuous variable were entered in the analysis separately.

∗ *P* < .05. BMI = body mass index, CI = confidence interval, eGFR = estimated glomerular filtration rate, FGF21 = fibroblast growth factor 21, HbA1c = glycated hemoglobin A1c, HOMA-IR = homeostatic model assessment of insulin resistance, LDL-c = low-density lipoprotein cholesterol, OR = odds ratio, SBP = systolic blood pressure, TG = triglycerides, UA = uric acid, UACR = urinary albumin/creatinine ratio.

### 3.2 Longitudinal study

In the longitudinal study consisting of a total of 2402 participants with baseline eGFR ≥60 mL/min/1.73 m^2^, 785 (32.7%) individuals had a rapid eGFR decline, defined as a decline in eGFR that exceeded 3.3%/year. The participants with eGFR decline were older in age, had higher blood pressure, lower fasting insulin, and HOMA-IR, but a higher prevalence of diabetes, higher UACR, lower SCr, and higher eGFR at baseline than those without eGFR decline. Serum FGF21 levels were not different between patients with and without an eGFR decline. Baseline BMI, HbA1c, FPG, lipid profiles, and UA levels were not different between the 2 groups. The median duration of follow-up was also comparable between the groups with and without eGFR decline (Table 3).

**Table 3:** Baseline characteristics by eGFR decline at follow-up in a total of 2402 participants.

	eGFR decline	Without eGFR decline	*P*
N	785	1617	
Age, y^∗^	49.8 ± 11.8	48.1 ± 11.5	.001
Male, n (%)^†^	361 (46.0)	710 (43.9)	.336
BMI, kg/m^2^^∗^	26.0 ± 3.8	26.2 ± 3.7	.411
SBP, mmHg^∗^	130.3 ± 17.6	128.6 ± 16.4	.019
DBP, mmHg^∗^	85.7 ± 11.9	84.5 ± 11.0	.010
HbA1c (%)^∗^	5.7 ± 1.0	5.7 ± 0.9	.438
FPG, mmol/L^∗^	5.9 ± 1.6	5.9 ± 1.7	.941
Fasting insulin, mU/L^‡^	6.8 (4.6–10.3)	7.3 (4.6–11.2)	.017
HOMA-IR^‡^	1.7 (1.1–2.7)	1.8 (1.2–2.9)	.046
Diabetes, n (%)^†^	136 (17.3)	217 (13.4)	.011
ALT, U/L^‡^	19 (15–27)	19 (15–27)	.647
AST, U/L^‡^	21 (18–25)	21 (18–24)	.222
TC, mmol/L^‡^	4.9 (4.3–5.5)	4.9 (4.3–5.5)	.583
TG, mmol/L^‡^	1.2 (0.8–1.9)	1.2 (0.8–1.8)	.137
LDL-C, mmol/L^∗^	2.8 ± 0.8	2.8 ± 0.8	.207
HDL-C, mmol/L^∗^	1.2 ± 0.3	1.2 ± 0.3	.918
UA, μmol/L^‡^	270 (221–323)	269 (224–327)	.686
UACR, mg/g^‡^	6.7 (2.3–18.6)	5.8 (2.2–13.9)	.011
SCr, μmol/L^‡^	53.0 (44.0–64.0)	58.0 (51.0–69.0)	<.001
eGFR (mL/min/1.73m^2^)^∗^	111.4 ± 15.0	107.4 ± 12.5	<.001
FGF21, pg/mL^‡^	82.6 (60.7–115.8)	82.8 (57.3–117.8)	.488
Duration of follow-up, mo^‡^	12 (11–14)	12 (11–14)	.617

eGFR decline is defined as that the declining rate of eGFR exceeds 3.3% per year.

∗ Variables were presented as the mean ± standard deviation.

† Variables were presented as frequency (%).

‡ Variables were presented as the median (interquartile range). ALT = alanine aminotransferase, AST = aspartate aminotransferase, BMI = body mass index, DBP = diastolic blood pressure, eGFR = estimated glomerular filtration rate, FGF21 = fibroblast growth factor 21, FPG = fasting plasma glucose, HbA1c = glycated hemoglobin A1c, HDL-c = high-density lipoprotein cholesterol, HOMA-IR = homeostatic model assessment of insulin resistance, LDL-c = low-density lipoprotein cholesterol, SBP = systolic blood pressure, SCr = serum creatinine, TC = total cholesterol, TG = triglycerides, UA = uric acid, UACR = urinary albumin/creatinine ratio

In the logistic regression analysis, only the second quartile of FGF21 was associated with an increased risk of eGFR decline (models 1 and 2 in Table 4). However, FGF21 as a continuous variable or the upper quartiles of FGF21 were not associated with increased odds of eGFR decline. Age and baseline eGFR were independent risk factors for the decline in eGFR (model 2 in Table 4).

**Table 4:** Associating factors for the decline of eGFR at follow-up (n = 2402).

	Model1-OR (95% CI)	Model2-OR (95% CI)
Age	1.013 (1.005–1.020)^∗^	1.098 (1.082–1.113)^∗^
Female vs male	0.920 (0.775–1.093)	0.698 (0.579–0.842)^∗^
BMI	0.990 (0.967–1.013)	
SBP	1.003 (0.997–1.008)	
Baseline eGFR	1.098 (1.083–1.112)^∗^	1.103 (1.088–1.118)^∗^
HbA1c	1.004 (0.918–1.099)	
HOMA-IR	0.953 (0.909–0.998)^∗^	0.978 (0.933–1.025)
TG	1.050 (0.981–1.123)	
LDL-C	0.902 (0.808–1.008)	
UA	0.999 (0.998–1.001)	
UACR	1.002 (1.001–1.002)^∗^	1.002 (1.001–1.003)
FGF21	1.000 (0.998–1.001)	
Quartiles of FGF21		
Q1 (FGF21 <58.50)	1.000	1.000
Q2 (82.67 > FGF21 ≥ 58.50)	1.301 (1.020–1.659)^∗^	1.329 (1.027–1.720)^∗^
Q3 (117.28 > FGF21 ≥ 82.67)	1.132 (0.884–1.449)	1.234 (0.948–1.607)
Q4 (FGF21 ≥ 117.28)	0.964 (0.745–1.247)	1.215 (0.922–1.003)

Model 1: adjusted for age and sex. Model2: adjusted for all the statistically significant variables in Model1. Quartiles of FGF21 and FGF21 as a continuous variable were entered in the analysis separately.

∗ *P* < .05. BMI = body mass index, CI = confidence interval, eGFR = estimated glomerular filtration rate, FGF21 = fibroblast growth factor 21, FPG = fasting plasm glucose, HbA1c = glycated hemoglobin A1c, HOMA-IR = homeostatic model assessment of insulin resistance, LDL-c = low-density lipoprotein cholesterol, OR = odds ratio, SBP = systolic blood pressure, TG = triglycerides, UA = uric acid, UACR = urinary albumin/creatinine ratio.

## 4 Discussion

The present study indicated an independent association between higher FGF21 levels and decreased eGFR in the cross-sectional baseline analysis in a population-based survey. In a longitudinal study with a median follow-up of 12 months, baseline FGF21 level was not associated with eGFR decline.

Previous cross-sectional studies have found that FGF21 levels are increased in patients with CKD and end-stage renal disease receiving hemodialysis or peritoneal dialysis.^[16–19]^ A negative association between eGFR and circulating FGF21 has also been found in participants without apparent kidney disease in both the community-dwelling population and patients with T2D.^[9,20]^ The result of the independent association of increased FGF21 and decreased eGFR (Table 2) in the present study was consistent with these previous studies.

Fibroblast growth factor 21 is a metabolic regulator with various benefits, not only in the metabolism of glucose and lipids. It is mainly expressed in the liver, induced by peroxisome proliferator-activated receptor-α. The function of this protein was first demonstrated in animal studies as the plasma glucose and metabolic state of diabetic mice and monkeys were improved with the administration of recombinant FGF21.^[21,22]^ However, the mechanism of action of FGF21 and kidney function have not been elucidated. Some evidence suggests that FGF21 could protect against renal fibrosis, which is often found in patients with CKD. A study found that FGF21 may play a role in the autophagic degradation of lipid droplets in kidney proximal tubular cells during prolonged starvation for energy homeostasis.^[10]^ Another study showed that FGF21 could prevent hyperglycemia-induced fibrogenesis in renal mesangial cells.^[11]^ In a murine model of diabetic nephropathy, FGF21 was found to reduce proteinuria and ameliorate morphological glomerular abnormalities.^[23]^ In addition, a study indicated that fenofibrate (a peroxisome proliferator-activated receptor-α agonist) treatment prevented renal damage in mice with T1D by up-regulating FGF21.^[24]^

Although FGF21 is beneficial to the kidney in animal studies, circulating FGF21 levels are increased in patients with decreased eGFR. The reasons for this are as follows. First, decreased glomerular filtration in CKD may directly lead to an increase in circulating FGF21 because FGF21 is a small molecule protein (approximately 21-kD) which can cross the glomerular capillary.^[25]^ However, FGF21 levels are still elevated in patients undergoing hemodialysis.^[16]^ Second, circulating FGF21 levels are usually elevated in metabolic disorders, including obesity and T2D.^[4]^ This is believed to be a compensatory response to metabolic stress in pathophysiological situations. As metabolic disorders are also risk factors for CKD, they may contribute to the elevation of FGF21 levels in patients with CKD.^[5]^

In the present study, the correlation between circulating FGF21 and UACR was only present in the subgroup of patients without diabetes (*P* = .048, presented in the supplementary table, http://links.lww.com/MD2/A753). Moreover, we did not find a positive association between FGF21 and BMI, HbA1c, and HOMA-IR, as reported in a previous study.^[4]^ Otherwise, SBP, AST, and LDL-c were positively correlated with serum FGF21 levels (Supplemental Table, http://links.lww.com/MD2/A753), in accordance with previous studies. In fact, although FGF21 has been considered as a biomarker for multiple metabolic disorders,^[1]^ the inconsistent association of circulating FGF21 and characteristics related to insulin resistance has also been demonstrated in some other cross-sectional studies.^[20,26]^ Despite the inconsistency in FGF21 and these metabolic markers, the association between FGF21 and renal function was independent of hypertension, diabetes, lipid profile, insulin resistance, and BMI in the present study and consistent with previous studies in different populations.^[16,18,19]^ Therefore, this suggests that renal function should be considered in all studies of FGF21 physiology as a strong associating factor.

Longitudinal studies in patients with T2D demonstrated a predictive effect of FGF21 on the progression of kidney diseases, represented as eGFR decline to new stages of CKD^[6]^ or new onset of diabetic nephropathy.^[7]^ A study conducted in 1700 Asian patients with T2Dfollowed for a mean of 6.3 years only found an association between FGF21 and end-stage renal disease in women. Furthermore, elevated FGF21 levels were shown to be an independent predictor of all-cause mortality in patients receiving chronic hemodialysis.^[27]^ In the present study of the general population, excluding those with eGFR <60 mL/min/1.73 m^2^, we did not determine an association between FGF21 and eGFR decline followed for a median of one year (Table 4). This is consistent with a recent multiethnic study consisted of 5724 participants free of cardiovascular disease followed for 10 years.^[9]^ In that study, the associations of FGF21 with neither eGFR decline nor UACR progression were detected in the longitudinal analysis, although FGF21 was independently associated with decreased eGFR in the cross-sectional part of the study.^[9]^ This may be because the decline in eGFR in relatively healthy people is slow, and circulating FGF21 has many influencing factors. Further investigations with new indicators of renal function and control of more influencing factors are needed.

The strengths of the present study are that it is based on a general population-based cohort with good quality control. To avoid potential bias in population selection, we used a stratified random 2-stage cluster sampling process based on natural age and sex distribution in both urban and rural communities. The overall response rate was 66.9%. Furthermore, it contains a longitudinal study with permission to assess causal relationships. This study adds new information to the association of circulating FGF21 with kidney function and the progression of kidney function in the general population.

This study had some limitations. First, the follow-up duration was short. The progression of kidney function in this study was defined as a decline in eGFR that exceeded 3.3% per year, calculated by only 1 follow-up visit. Because of the characteristics of the population and short duration of follow-up, we cannot judge the substantial renal outcome with indices such as a 30% to 50% eGFR decline, or a doubling of SCr. We attempted to use the other criteria, including an eGFR decline of 5% or 5 mL/min/year, and confirmed the results consistent with the current analysis. Second, eGFR was calculated with the EPI equation using only SCr without cystatin C. This equation was generated in Whites but not in Asians. Therefore, there may be a lack of accurate representation of kidney function in this population. Third, circulating FGF21 was also influenced by other factors including activity levels, smoking habits, and so on. Although all the blood samples were collected in the fasting state in the morning, other potential factors may still influence FGF21 levels and could not be adjusted because of incomplete information.

## 5 Conclusions

Increased serum FGF21 levels were independently associated with lower eGFR in this population-based study, whereas baseline FGF21 did not predict the progression of eGFR over a median follow-up of 12 months in this nonmedicated general population.

## Acknowledgments

The authors thank the research teams from the Endocrinology and Metabolism Departments of Beijing Pinggu Hospital and Peking University People's Hospital for their contribution to the field survey and data collection.

## Author contributions

R.Z. wrote the manuscript. Y.L. conducted baseline and follow-up investigations. X.Z. and L.J. designed and directed the study F.Z. and M.L. sorted the serum samples and performed the tests. S.Z. and X.Z. performed the fieldwork. X.W. helped with the analysis. All authors reviewed the manuscript.

**Conceptualization:** Xianghai Zhou, Linong Ji.

**Data curation:** Yufeng Li, Xianghai Zhou, Fang Zhang, Meng Li, Simin Zhang, Xiuying Zhang, Xin Wen.

**Formal analysis:** Rui Zhang, Meng Li, Simin Zhang.

**Funding acquisition:** Rui Zhang, Xianghai Zhou, Linong Ji.

**Investigation:** Yufeng Li, Fang Zhang, Xiuying Zhang, Xin Wen, Linong Ji.

**Methodology:** Linong Ji.

**Project administration:** Yufeng Li, Xianghai Zhou, Fang Zhang, Meng Li, Simin Zhang, Xiuying Zhang, Xin Wen.

**Software:** Xin Wen.

**Supervision:** Yufeng Li, Xianghai Zhou, Xiuying Zhang, Linong Ji.

**Writing – original draft:** Rui Zhang.

**Writing – review & editing:** Xianghai Zhou.

## References

[1] Woo YC Xu A Wang Y. Fibroblast growth factor 21 as an emerging metabolic regulator: clinical perspectives. Clin Endocrinol (Oxf) 2013;78:489–96. 23134073 10.1111/cen.12095

[2] Charles ED Neuschwander-Tetri BA Pablo Frias J. Pegbelfermin (BMS-986036), PEGylated FGF21, in patients with obesity and type 2 diabetes: results from a randomized phase 2 study. Obesity (Silver Spring) 2019;27:41–9. 30520566 10.1002/oby.22344 PMC6587787

[3] Gaich G Chien JY Fu H. The effects of LY2405319, an FGF21 analog, in obese human subjects with type 2 diabetes. Cell Metab 2013;18:333–40. 24011069 10.1016/j.cmet.2013.08.005

[4] Zhang X Yeung DC Karpisek M. Serum FGF21 levels are increased in obesity and are independently associated with the metabolic syndrome in humans. Diabetes 2008;57:1246–53. 18252893 10.2337/db07-1476

[5] Anuwatmatee S Tang S Wu BJ. Fibroblast growth factor 21 in chronic kidney disease. Clin Chim Acta 2019;489:196–202. 29108880 10.1016/j.cca.2017.11.002

[6] Lee CH Hui EY Woo YC. Circulating fibroblast growth factor 21 levels predict progressive kidney disease in subjects with type 2 diabetes and normoalbuminuria. J Clin Endocrinol Metab 2015;100:1368–75. 25625802 10.1210/jc.2014-3465

[7] Ong KL Januszewski AS O’Connell R. Relationship of fibroblast growth factor 21 with baseline and new on-study microvascular disease in the Fenofibrate Intervention and Event Lowering in Diabetes study. Diabetologia 2015;58:2035–44. 26055067 10.1007/s00125-015-3652-2

[8] Liu JJ Liu S Choo RWM. Sex modulates the association of fibroblast growth factor 21 with end-stage renal disease in Asian people with Type 2 diabetes: a 6.3-year prospective cohort study. Diabet Med 2018;35:880–6. 29653030 10.1111/dme.13641

[9] Anuwatmatee S Allison MA Shlipak MG. Relationship of fibroblast growth factor 21 with kidney function and albuminuria: multi-ethnic study of atherosclerosis. Nephrol Dial Transplant 2019;34:1009–16. 29771383 10.1093/ndt/gfy120 PMC6545465

[10] Minami S Yamamoto T Takabatake Y. Lipophagy maintains energy homeostasis in the kidney proximal tubule during prolonged starvation. Autophagy 2017;13:1629–47. 28813167 10.1080/15548627.2017.1341464 PMC5640178

[11] Li S Guo X Zhang T. Fibroblast growth factor 21 ameliorates high glucose-induced fibrogenesis in mesangial cells through inhibiting STAT5 signaling pathway. Biomed Pharmacother 2017;93:695–704. 28692941 10.1016/j.biopha.2017.06.100

[12] Zou X Li Y Cai X. Decreased glycemic difference between diabetes and nondiabetes in the elderly leads to the reduced diagnostic accuracy of hemoglobin a1c for diabetes screening in an aged Chinese population. Diabetes Technol Ther 2016;18:226–32. 26894547 10.1089/dia.2015.0353

[13] American Diabetes Association. 2. Classification and diagnosis of diabetes: standards of medical care in diabetes-2019. Diabetes Care 2019;42: (suppl 1): S13–28. 30559228 10.2337/dc19-S002

[14] Inker LA Schmid CH Tighiouart H. Estimating glomerular filtration rate from serum creatinine and cystatin C. N Engl J Med 2012;367:20–9. 22762315 10.1056/NEJMoa1114248 PMC4398023

[15] Seo DH Kim SH Song JH. Presence of carotid plaque is associated with rapid renal function decline in patients with type 2 diabetes mellitus and normal renal function. Diabetes Metab J 2019;43:840–53. 30877715 10.4093/dmj.2018.0186 PMC6943261

[16] Stein S Bachmann A Lossner U. Serum levels of the adipokine FGF21 depend on renal function. Diabetes Care 2009;32:126–8. 18840768 10.2337/dc08-1054 PMC2606845

[17] Han SH Choi SH Cho BJ. Serum fibroblast growth factor-21 concentration is associated with residual renal function and insulin resistance in end-stage renal disease patients receiving long-term peritoneal dialysis. Metabolism 2010;59:1656–62. 20423749 10.1016/j.metabol.2010.03.018

[18] Lin Z Zhou Z Liu Y. Circulating FGF21 levels are progressively increased from the early to end stages of chronic kidney diseases and are associated with renal function in Chinese. PLoS One 2011;6:e18398. 21525989 10.1371/journal.pone.0018398 PMC3078122

[19] Crasto C Semba RD Sun K. Serum fibroblast growth factor 21 is associated with renal function and chronic kidney disease in community-dwelling adults. J Am Geriatr Soc 2012;60:792–3. 22494291 10.1111/j.1532-5415.2011.03879.x PMC3325515

[20] Jian WX Peng WH Jin J. Association between serum fibroblast growth factor 21 and diabetic nephropathy. Metabolism 2012;61:853–9. 22136913 10.1016/j.metabol.2011.10.012

[21] Kharitonenkov A Shiyanova TL Koester A. FGF-21 as a novel metabolic regulator. J Clin Invest 2005;115:1627–35. 15902306 10.1172/JCI23606 PMC1088017

[22] Kharitonenkov A Wroblewski VJ Koester A. The metabolic state of diabetic monkeys is regulated by fibroblast growth factor-21. Endocrinology 2007;148:774–81. 17068132 10.1210/en.2006-1168

[23] Kim HW Lee JE Cha JJ. Fibroblast growth factor 21 improves insulin resistance and ameliorates renal injury in db/db mice. Endocrinology 2013;154:3366–76. 23825123 10.1210/en.2012-2276

[24] Cheng Y Zhang J Guo W. Up-regulation of Nrf2 is involved in FGF21-mediated fenofibrate protection against type 1 diabetic nephropathy. Free Radic Biol Med 2016;93:94–109. 26849944 10.1016/j.freeradbiomed.2016.02.002 PMC7446394

[25] Suassuna PGA de Paula RB Sanders-Pinheiro H. Fibroblast growth factor 21 in chronic kidney disease. J Nephrol 2019;32:365–77. 30430412 10.1007/s40620-018-0550-y PMC6483847

[26] Hindricks J Ebert T Bachmann A. Serum levels of fibroblast growth factor-21 are increased in chronic and acute renal dysfunction. Clin Endocrinol (Oxf) 2014;80:918–24. 24612017 10.1111/cen.12380

[27] Kohara M Masuda T Shiizaki K. Association between circulating fibroblast growth factor 21 and mortality in end-stage renal disease. PLoS One 2017;12:e0178971. 28582462 10.1371/journal.pone.0178971 PMC5459464

